# A Fragile Dignity Despite Their Rags and Tatters

**DOI:** 10.3201/eid2010.AC2010

**Published:** 2014-10

**Authors:** Byron Breedlove

**Affiliations:** Centers for Disease Control and Prevention, Atlanta, Georgia, USA

**Keywords:** art science connection, emerging infectious diseases, vector-borne diseases, poverty, art and medicine, Gustave Doré, Beggars of Burgos, A Fragile Dignity Despite Their Rags and Tatters, about the cover

**Figure:**
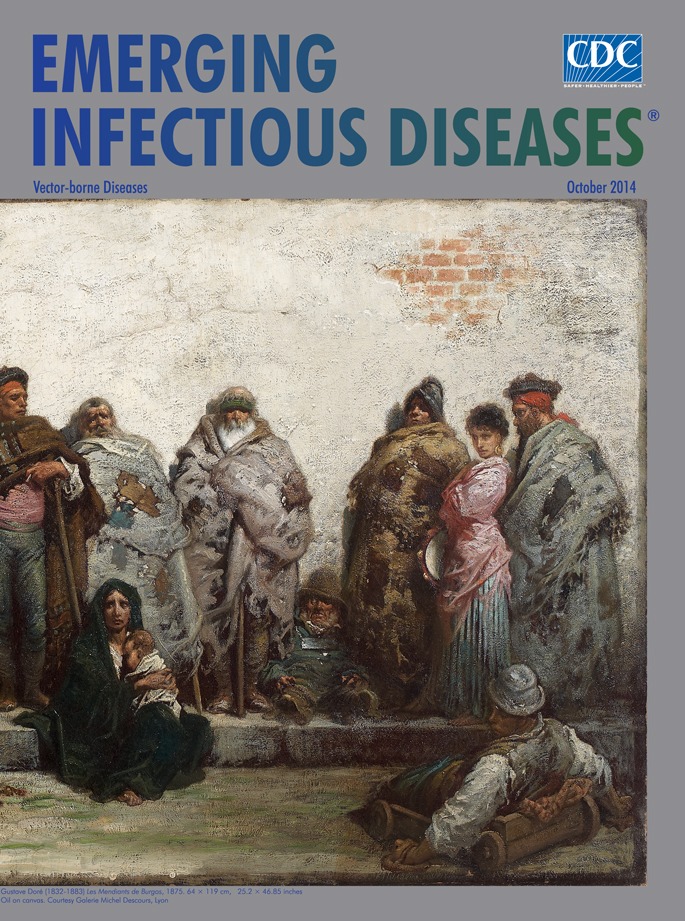
**Gustave Doré (1832–1883) Beggars of Burgos, 1875. Oil on canvas (64 × 119 cm).** Private collection, Courtesy of Galerie Michel Descours, Lyon, France.

Gustave Doré, born in Strasbourg, France, in 1832, was one of the most prodigious artists of the 19th century. According to Dan Malan, one of his biographers, his nearly 10,000 engravings and 3,000 book editions made him the most prolific and popular illustrator of all time. Doré also produced around 400 oil paintings, as well as watercolor landscapes, mixed media sketches, and sculptures. According to another Doré biographer, “The speed with which he drew was legendary and his output was as noteworthy for its quantity as for its quality.”

Vincent van Gogh referred to him as an “Artist of the People” because Doré directed his work to the masses through his popular literary folios. He worked as a caricaturist and professional illustrator when he was just 15 years of age. Doré illustrated texts by Dante, Rabelais, Perrault, Cervantes, Milton, Shakespeare, Hugo, Balzac, and Poe, plus the Bible. The publication of his engravings in Dante's *Inferno* in 1861 brought him international fame.

Doré contributed 180 engravings to the book *London: A Pilgrimage* (1872), written by Blanchard Jerrold. Though commercially successful, the book spurred criticism as Doré included stark images of the poverty and despair he encountered. The *Westminster Review* noted, for example, that “Dore gives us sketches in which the commonest, the vulgarest external features are set down.”

Throughout his career, Doré was sensitive to poverty, social misery, and human suffering. On this month’s cover image, Castilian beggars cluster before a whitewashed wall as though they have assembled for a portrait. Most are dressed in tattered blankets, threadbare shawls, and disheveled clothing though some wear colorful sashes and hats. A young mother, perhaps a recent widow, sits alone holding her infant. A crippled man lies in a small wooden wagon, his hands wrapped in leather or rags. Near the center, a tall man leans on walking sticks; one family with two small children stands to his left, while a couple with sprawl on the sidewalk with an infant and a dog. Others beggars group seen at the edges of the painting include a woman with tambourine and several men who may have once been soldiers or tradesmen fallen on hard times. Whatever their stories, Doré paints these beggars, bound by poverty and lassitude, as still having a fragile dignity.

Now, as then, the poorest people, such as those depicted on Doré’s cover painting, often live in the worst environments, are crowded together, lack adequate shelter, do not have clean water or sanitation, and suffer malnutrition—ideal circumstances for infectious disease transmission.
